# Association of obesity profiles and metabolic health status with liver injury among US adult population in NHANES 1999–2016

**DOI:** 10.1038/s41598-023-43028-7

**Published:** 2023-09-25

**Authors:** Jing Huang, Tian Gao, Huinan Zhang, Xing Wang

**Affiliations:** Department of Health and Management, Tangdu Hospital, Air Force Military Medical University, Xi’an, 710038 Shaanxi China

**Keywords:** Obesity, Metabolic disorders, Liver diseases

## Abstract

The combined effect of obesity and metabolic abnormalities on liver injury is unclear. Aiming to address this knowledge gap, this cross-sectional study was conducted among 16,201 US adults. Multiple linear regression and logistic regression analyses were conducted to assess the associations of obesity profiles, metabolic health status, and weight change with the levels of liver enzymes. The analysis revealed that general obesity and abdominal obesity were positively associated with the levels of liver enzymes and the prevalence of abnormal liver enzymes (*P* and *P*_trend_ < 0.05). The associations remained significant in both metabolically healthy and metabolically unhealthy subgroups. Additionally, the liver injury index levels of the metabolically unhealthy participants were higher than those of the metabolically healthy individuals within the non-obese, overweight/pre-abdominal obesity, and general/abdominal obesity subgroups (*P* and *P*_trend_ < 0.05). Furthermore, the subgroup characterized by general/abdominal obesity and metabolic dysfunction exhibited the most robust association with the liver injury index compared to all other subgroups examined. In addition, positive associations were observed between the 1-year and 10-year weight changes and the levels of liver injury indicators (*P* and *P*_trend_ < 0.05). In conclusion, this study demonstrates that both obesity and metabolic impairment are independently associated with liver injury, and their combined presence have an additional adverse effect on liver health. These findings underscore the importance of addressing both obesity and metabolic dysfunction in order to mitigate the risk of liver injury.

## Introduction

The liver is one of the main metabolic organs in the human body and participates in a series of important physiological functions, including the decomposition, synthesis, storage, and detoxification of compounds^1^. With the rapid progression of economic development and changes in lifestyle, liver disease has become a heavy health burden worldwide^2^. Deaths related to liver disease increased by 11.4% from 2012 to 2017, and accounts for approximately 2 million deaths each year^3^. Moreover, the number of years lived with disability due to liver disease also increased by 50.7% from 1990 to 2007, and by 34.8% from 2007 to 2017^4^. Consequently, the burden of liver disease has attracted extensive attention. Alongside viral hepatitis and alcohol consumption, nonalcoholic fatty liver disease (NAFLD) has emerged as a rapidly growing contributor to liver mortality and morbidity^3^. It is the most prevalent liver disorder and major cause for elevated liver enzymes in Asia^5^.

In addition to viruses, alcohol consumption, nutritional factors, and chemical toxins^6,7^, the adverse effects of obesity and metabolic dysfunction on the liver have been partially understood. A retrospective study including 767 participants suggested that obesity severity was positively associated with liver disease severity^8^. Furthermore, a large-population study using Mendelian randomization showed that the two major subtypes of obesity, general and abdominal obesity, which can be measured by body mass index (BMI) and waist circumference (WC), respectively, were causal factors for NAFLD and chronic liver disease in 228,466 women and 195,041men^9^. When comparing general obesity with abdominal obesity, the latter exhibited a closer association with type 2 diabetes, hypertension, dyslipidemia, cardiovascular disease, cancer, and all-cause mortality^10,11^. Both general obesity and abdominal obesity have been identified to be associated with liver injury in previous studies^12,13^, nevertheless, few studies have compared the contributions of these two obesity phenotypes to liver injury.

In addition to obesity, metabolic disorders including insulin resistance, dyslipidemia, and hypertension are also risk factors for liver disease^14–16^. A cross-sectional study involving 236 Mexican children and adolescents found that the prevalence of metabolic syndrome was strongly associated with elevated liver enzyme levels^17^. Additionally, the causal effects of obesity traits and metabolic diseases, including type 2 diabetes mellitus (T2DM) and hypertension, on the risk of NAFLD has been identified by a Mendelian randomization study^18^. Interactions exit between obesity and metabolic disorders^19^. Obesity could induce metabolic dysfunctions, most likely by inflammatory mechanisms^20,21^. But individuals with obesity may have limited or no features of poor metabolic health, which is referred to as metabolically healthy obesity (MHO)^22^. Recent research suggests that MHO might be a transient state associated with long-term health complications, including an increased risk of heart failure^23^. Though both obesity and metabolic dysfunctions have been implicated in liver injury, further studies are needed to explore the combined effects of different obesity phenotype and metabolic health status on liver injury.

In this study, we conducted cross-sectional and retrospective analyses using data of general adults in the United States from the 1999–2016 survey cycles of the National Health and Nutrition Examination Survey (NHANES). Liver injury was assessed based on the levels of liver enzymes, including alanine aminotransferase (ALT), aspartate aminotransferase (AST), alkaline phosphatase (ALP), gamma-glutamyl transferase (GGT), and the AST/ALT ratio. General obesity and abdominal obesity were assessed by BMI and WC, respectively. Metabolic health status was assessed by fasting serum triglyceride (TG), high-density lipoprotein cholesterol (HDL-C), systolic blood pressure (SBP), diastolic blood pressure (DBP), fasting blood glucose (FBG), and a general health questionnaire. The objectives of this study were to assess the associations of different obesity phenotypes and metabolic health status with liver injury, and to evaluate the combined effects of obesity and metabolically unhealthy status.

## Materials and methods

### Study population

The study participants were sourced from the NHANES, as detailed in existing studies^24,25^. Briefly, US civilians were recruited using a complex, multistage probability design, conducted by Centers for Disease Control and Prevention (CDC)’s National Center for Health Statistics (NCHS). Comprehensive data collection, including questionnaire interviews, physical examinations, and laboratory tests have been conducted biennially since 1999. The study protocol was approved by the NCHS Research Ethics Review Board, and all participants signed informed consent forms. All methods were performed in accordance with the relevant guidelines and regulations.

For the present analyses, the study focused on adults aged 20–85, utilizing data from NHANES cycles conducted between 1999 and 2016. This time frame was chosen due to inconsistencies observed in questionnaire content, specifically regarding alcohol consumption, which varied since 2017. Additionally, the potential influence of the COVID-19 pandemic starting in 2019 necessitated a focus on pre-pandemic data. After excluding participants without physical examinations or laboratory tests, pregnant individuals, and those infected with hepatitis B or C, a total of 16,201 participants was included in the cross-sectional analyses. Subsequently, after further exclusion of participants lacking retrospective weight data from 10 years ago, 11,677 participants were available for retrospective analyses. The dataset used for this analysis encompasses NHANES data from 1999 to 2016. The details of the program, collection procedures, and data files are publicly available at NHANES website https://www.cdc.gov/nchs/nhanes/.

### Assessment of obesity and metabolically healthy status

In NHANES, weight, height, and WC were measured by expert anthropometrists to the nearest 0.1 kg and 0.1 cm, respectively, and BMI was calculated by dividing weight (kg) by the squared of height (m^2^). In this study, non-general obesity was defined as BMI < 25.0 kg/m^2^, overweight was defined as 25.0 kg/m^2^ ≤ BMI < 30.0 kg/m^2^, and general obesity was defined as BMI ≥ 30.0 kg/m^2^. Non-abdominal obesity was defined as WC < 94.0 cm in men or < 80.0 cm in women, pre-abdominal obesity was defined as 94.0 cm ≤ WC < 102.0 cm in men or 80.0 cm ≤ WC < 88.0 cm in women, and abdominal obesity was defined as WC ≥ 102.0 cm in men or WC ≥ 88.0 cm in women^26^. In addition, 1-year and 10-year weight changes were calculated by subtracting the self-reported weights 1 and 10 years prior from the current weight.

In this study, metabolically healthy status was defined as: serum TG ≤ 1.7 mmol/L, HDL-C > 1.0 mmol/L in men or > 1.3 mmol/L in women, SBP ≤ 130 mmHg, DBP ≤ 85 mmHg, FBG ≤ 6.1 mmol/L, and no drug treatment for dyslipidemia, diabetes, or hypertension^22^. Otherwise, individuals were classified as metabolically unhealthy individuals.

### Assessment of liver injury

In NHANES, liver enzymes, including ALT, AST, ALP, and GGT were measured during different cycles using specific analyzers. Specifically, the Hitachi Model 704 multichannel analyzer was employed during the 1999–2002 cycles, followed by the Beckman Synchron LX20 during the 2003–2006 cycles, and the Beckman UniCel^®^ DxC800 Synchron during the 2007–2016 cycles, all measured by medical technologists. Rigorous quality control and assurance measures were implemented in compliance with the 1988 Clinical Laboratory Improvement Act. For values of liver enzymes falling below the lower detection limit, an adjustment was made by replacing them with a value equal to the detection limit divided by the square root of the two. In this study, abnormal liver enzymes were defined in line with prior research: ALT > 47.0 IU/L in men or > 30.0 in women, AST > 33.0 IU/L in men and women, ALP > 113.0 IU/L in men and women, GGT > 65.0 IU/L in men and > 36.0 IU/L in women^27^. In addition, the AST/ALT ratio, calculated by dividing AST by ALT, is also an important indicator of liver injury.

### Assessment of covariates

In NHANES, demographic and lifestyle data, including age, gender, race, educational qualification, physical exercise, annual household income, smoking status, and alcohol consumption, were collected using standard questionnaires by trained investigators. In this study, race categories included Mexican Americans, other Hispanics, non-Hispanic whites, non-Hispanic blacks, and other races; educational qualifications were categorized as less than 9th grade, 9–11th grade, high school graduate, associate degree, and college graduate or above; physical exercise was divided into “yes” (defined as vigorous activities that caused heavy sweating or large increases in breathing or heart rate) and “no”; annual household income was divided into high (above the median for each cycle) and low; smoking status was divided into “yes” and “no”; and alcohol consumption was dichotomized as “yes” (drinking at least once a week over the past 12 months) and “no”.

### Statistical analysis

In order to address their right-skewed distribution, the levels of liver enzymes and the AST/ALT ratio were subjected to a natural logarithmic transformation. Analysis of Variance, Kruskal–Wallis test, and chi-square test were used to compare the differences in basic characteristics among participants with different general obesity statuses or abdominal obesity statuses for symmetrically distributed variables, right-skewed distributed variables, and categorical variables, respectively. Multiple linear regression and logistic regression analyses were conducted to estimate the associations of general/abdominal obesity with liver enzyme levels as well as the prevalence of abnormal liver enzymes. Potential confounders such as age, gender, race, educational qualification, physical exercise, annual household income, smoking status, alcohol consumption, and batch (survey cycle) were included in the analysis and the results were adjusted for the aforementioned covariates. The percentage of R^2^ change in the regression analyses was calculated to compare the relative contributions of general obesity and abdominal obesity to adverse effects on liver injury. The participants were further stratified into six subgroups according to general/abdominal obesity status and metabolic health status to assess the combined effects of obesity and metabolic disorders on the liver, with the subgroup of non-general/abdominal obesity and metabolically healthy status as the reference. In retrospective analyses, the associations of 1-year and 10-year weight changes with the levels of liver enzymes and the prevalence of abnormal liver enzymes were evaluated using multiple linear regression and logistic regression analyses.

All *P* values were two-sided with a statistical significance level of 0.05, and survey-weighted multiple linear and logistical regression analyses were performed with R software (version 4.2.0, R Foundation for Statistical Computing, Austria).

## Results

### Basic characteristics

As presented in Table 1, the mean age of 16,201 participants was 49.6 (standard deviation, 17.8) years, with 8119 (50.1%) being male. Participants were categorized into two main groups based on major subtypes of obesity: general obesity and abdominal obesity, which were determined by BMI and WC, respectively. Within the general obesity group, the proportions for non-general obesity, overweight, and general obesity subgroups were 31.1%, 34.5%, and 34.4%, respectively. Within the abdominal obesity group, the proportions for non-abdominal, pre-abdominal, and abdominal obesity subgroups were 25.2%, 20.5%, and 54.2%, respectively. Smoking and drinking rates were 17.1% and 42.2%, respectively. Abnormal ALT, AST, ALP, or GGT were observed in less than 10% of the population, while over 70% of the participants were metabolically unhealthy. Ratios of participants with abnormal liver enzyme levels and unhealthy metabolism increased with the degree of obesity, while AST/ALT decreased (all *P* < 0.05). These trends were observed in both general obesity and abdominal obesity.

**Table 1:** Characteristics of survey participants.

Characteristics	Total	General obesity				Abdominal obesity			
		Non-general obesity	Overweight	General obesity	*P*	Non-abdominal obesity	Pre-abdominal obesity	Abdominal obesity	*P*
N	16,201	5040 (31.1)	5589 (34.5)	5572 (34.4)		4088 (25.2)	3328 (20.5)	8785 (54.2)	
Age, years	49.6 ± 17.8	47.0 ± 19.2	51.3 ± 17.7	50.3 ± 16.4	< 0.001	42.4 ± 17.8	49.7 ± 17.6	53.0 ± 16.9	< 0.001
Gender					< 0.001				< 0.001
Male	8119 (50.1)	2400 (29.6)	3201 (39.4)	2518 (31.0)		2821 (34.7)	1871 (23.0)	3427 (42.2)	
Female	8082 (49.9)	2640 (32.7)	2388 (29.5)	3054 (37.8)		1267 (15.7)	1457 (18.0)	5358 (66.3)	
Race					< 0.001				< 0.001
Mexican American	2798 (17.3)	600 (21.4)	1129 (40.3)	1069 (38.2)		573 (20.5)	626 (22.4)	1599 (57.1)	
Other Hispanic	1415 (8.7)	344 (24.3)	562 (39.7)	509 (36.0)		312 (22.0)	308 (21.8)	795 (56.2)	
Non-Hispanic white	7656 (47.3)	2602 (34.0)	2627 (34.3)	2427 (31.7)		1822 (23.8)	1582 (20.7)	4252 (55.5)	
Non-Hispanic black	3053 (18.8)	786 (25.7)	918 (30.1)	1349 (44.2)		808 (26.5)	487 (16.0)	1758 (57.6)	
Other races	1279 (7.9)	708 (55.4)	353 (27.6)	218 (17.0)		573 (44.8)	325 (25.4)	381 (29.8)	
Educational qualification					< 0.001				< 0.001
Less than 9th grade	1896 (11.7)	473 (24.9)	745 (39.3)	678 (35.8)		384 (20.3)	394 (20.8)	1118 (59.0)	
9–11th grade	2368 (14.6)	694 (29.3)	786 (33.2)	888 (37.5)		583 (24.6)	448 (19.0)	1337 (56.5)	
High school graduate	3704 (22.9)	1073 (29.0)	1294 (34.9)	1337 (36.1)		889 (24.0)	736 (19.9)	2079 (56.1)	
Associate degree	4562 (28.2)	1382 (30.3)	1516 (33.2)	1664 (36.5)		1126 (24.7)	877 (19.2)	2559 (56.1)	
College or above	3671 (22.7)	1418 (38.6)	1248 (34.0)	1005 (27.4)		1106 (30.1)	873 (23.8)	1692 (46.1)	
Physical exercise					< 0.001				< 0.001
Yes	3850 (23.8)	1263 (32.8)	1386 (36.0)	1201 (31.2)		1285 (33.4)	821 (21.3)	1744 (45.3)	
No	12,351 (76.2)	3777 (30.6)	4203 (34.0)	4371 (35.4)		2803 (22.7)	2507 (20.3)	7041 (57.0)	
Annual household income				<0.001	<0.001				< 0.001
High	7329 (45.2)	2382 (32.5)	2588 (35.3)	2359 (32.2)		1951 (26.6)	1670 (22.8)	3708 (50.6)	
Low	8872 (54.8)	2658 (30.0)	3001 (33.8)	3213 (36.2)		2137 (24.1)	1658 (18.7)	5077 (57.2)	
Smoking status					< 0.001				< 0.001
Yes	2775 (17.1)	1104 (39.8)	852 (30.7)	819 (29.5)		917 (33.0)	566 (20.4)	1292 (46.6)	
No	13,426 (82.9)	3936 (29.3)	4737 (35.3)	4753 (35.4)		3171 (23.6)	2762 (20.6)	7493 (55.8)	
Alcohol consumption					< 0.001				< 0.001
Yes	6834 (42.2)	2279 (33.3)	2412 (35.3)	2143 (31.4)		1952 (28.6)	1522 (22.3)	3360 (49.2)	
No	9367 (57.8)	2761 (29.5)	3177 (33.9)	3429 (36.6)		2136 (22.8)	1806 (19.3)	5425 (57.9)	
ALT, IU/L	21.0 (16.0, 28.0)	19.0 (15.0, 24.0)	22.0 (17.0, 29.0)	23.0 (17.0, 31.0)	< 0.001	20.0 (16.0, 26.0)	21.0 (16.0, 29.0)	21.0 (17.0, 29.0)	< 0.001
AST, IU/L	23.0 (19.0, 27.0)	22.0 (19.0, 26.0)	23.0 (20.0, 27.0)	23.0 (19.0, 28.0)	< 0.001	23.0 (20.0, 27.0)	23.0 (19.0, 27.0)	22.0 (19.0, 27.0)	< 0.001
ALP, IU/L	66.0 (54.0, 81.0)	62.0 (51.0, 78.0)	66.0 (55.0, 80.0)	69.0 (57.0, 84.0)	< 0.001	62.0 (51.0, 77.0)	65.0 (53.0, 78.0)	69.0 (57.0, 84.0)	< 0.001
GGT, IU/L	20.0 (14.0, 30.0)	17.0 (12.0, 24.0)	21.0 (15.0, 31.0)	23.0 (17.0, 34.0)	< 0.001	18.0 (13.0, 26.0)	20.0 (14.0, 29.0)	21.0 (15.0, 32.0)	< 0.001
AST/ALT	1.1 (0.9, 1.3)	1.2 (1.0, 1.4)	1.1 (0.9, 1.3)	1.0 (0.8, 1.2)	< 0.001	1.2 (1.0, 1.4)	1.1 (0.9, 1.3)	1.0 (0.9, 1.2)	< 0.001
Abnormal ALT	1565 (9.7)	282 (18.0)	512 (32.7)	771 (49.3)	< 0.001	212 (13.5)	297 (19.0)	1056 (67.5)	< 0.001
Abnormal AST	1606 (9.9)	422 (26.3)	538 (33.5)	646 (40.2)	< 0.001	407 (25.3)	338 (21.0)	861 (53.6)	0.838
Abnormal ALP	643 (4.0)	168 (26.1)	217 (33.7)	258 (40.1)	0.003	118 (18.4)	108 (16.8)	417 (64.9)	< 0.001
Abnormal GGT	1541 (9.5)	328 (21.3)	510 (33.1)	703 (45.6)	< 0.001	225 (14.6)	271 (17.6)	1045 (67.8)	< 0.001
Metabolically unhealthy status	11,428 (70.5)	2635 (23.1)	4100 (35.9)	4693 (41.1)	< 0.001	1941 (17.0)	2261 (19.8)	7226 (63.2)	< 0.001
Abnormal BP	5477 (33.8)	1341 (24.5)	1924 (35.1)	2212 (40.4)	< 0.001	945 (17.3)	1070 (19.5)	3462 (63.2)	< 0.001
Abnormal FBG	3827 (23.6)	600 (15.7)	1290 (33.7)	1937 (50.6)	< 0.001	440 (11.5)	626 (16.4)	2761 (72.1)	< 0.001
Abnormal TG	4267 (23.6)	716 (16.8)	1639 (38.4)	1912 (44.8)	< 0.001	564 (13.2)	801 (18.8)	2902 (68.0)	< 0.001
Abnormal HDL-C	4452 (27.5)	774 (17.4)	1480 (33.2)	2198 (49.4)	< 0.001	508 (11.4)	776 (17.4)	3168 (71.2)	< 0.001

Definition of abbreviations: *ALT* alanine aminotransferase, *AST* aspartate aminotransferase, *ALP* alkaline phosphatase, *GGT* gamma-glutamyl transferase, *BP* blood pressure, *FBG* fasting blood glucose, *TG* triglycerides, HDL-C high density lipoprotein cholesterol. Data are presented as mean (standard deviation), number (percentage), and median (quartile range) for symmetric distributed variables, categorical variables, and skewed distributed variables, respectively.

### Associations of general obesity and abdominal obesity with liver injury

The associations of general obesity with liver enzyme levels as well as the prevalence of abnormal liver enzymes are presented in Table 2. After adjusting for potential covariates, the levels of ALT, AST, ALP, and GGT significantly increased as degrees of general obesity increased, while the AST/ALT ratio decreased significantly (all *P* and *P*_trend_ < 0.05). With the non-general obesity subgroup as a reference, the levels of ALT, AST, ALP, and GGT in the general obesity subgroup increased by 26.2% (24.0%, 28.4%), 3.4% (1.8%, 5.0%), 10.0% (8.5%, 11.5%), and 34.7% (32.0%, 37.3%), respectively. A similar trend was also observed for the presence of abnormal liver enzymes. General obesity was positively associated with liver enzyme levels as well as the presence of abnormal liver test (all *P*_trend_ < 0.05).

**Table 2:** Associations of general obesity with liver injury.

Indicators of liver injury	β/OR (95% *CI*)			*P*_trend_	Percentage of R^2^ change (%)
	Non-general obesity	Overweight	General obesity		
Continuous outcome					
ALT	0 (reference)	0.143 (0.122, 0.164)***	0.262 (0.240, 0.284)***	< 0.001	35.665
AST	0 (reference)	0.017 (0.002, 0.032)*	0.034 (0.018, 0.050)***	< 0.001	2.369
ALP	0 (reference)	0.053 (0.039, 0.067)***	0.100 (0.085, 0.115)***	< 0.001	16.795
GGT	0 (reference)	0.158 (0.131, 0.186)***	0.347 (0.320, 0.373)***	< 0.001	38.008
AST/ALT	0 (reference)	− 0.126 (− 0.140, − 0.112)***	− 0.228 (− 0.242, − 0.214)***	< 0.001	73.918
Categorical outcome					
Abnormal ALT	1 (reference)	2.149 (1.740, 2.654)***	3.707 (3.061, 4.489)***	< 0.001	147.263
Abnormal AST	1 (reference)	1.201 (0.988, 1.459)	1.683 (1.389, 2.039)***	< 0.001	20.768
Abnormal ALP	1 (reference)	0.991 (0.714, 1.376)	1.408 (1.033, 1.919)*	0.025	4.296
Abnormal GGT	1 (reference)	1.584 (1.309, 1.916)***	2.461 (2.070, 2.926)***	< 0.001	44.134

Models were adjusted for age, gender, race, educational qualification, physical exercise, annual household income, smoking status, alcohol consumption, and batch (survey cycles). *P*_trend_ was tested by including non-general obesity, overweight, and general obesity as 1, 2, and 3 (continuous variable) in models, respectively. *P < 0.05, **P < 0.01, ***P < 0.001.

As shown in Table 3, the levels of ALT, AST, ALP, and GGT as well as the prevalence of abdominal liver enzymes increased significantly from non-abdominal obesity to pre-abdominal obesity and finally to abdominal obesity (all *P*_trend_ < 0.05).

**Table 3:** Associations of Abdominal obesity with liver injury.

Indicators of liver injury	β/OR (95% *CI*)			*P*_trend_	Percentage of R^2^ change (%)
	Non-abdominal obesity	Pre-abdominal obesity	Abdominal obesity		
Continuous outcome					
ALT	0 (reference)	0.125 (0.101, 0.149)***	0.243 (0.220, 0.266)***	< 0.001	30.281
AST	0 (reference)	0.011 (− 0.005, 0.027)	0.024 (0.007, 0.041)**	0.005	1.233
ALP	0 (reference)	0.020 (0.003, 0.036)***	0.104 (0.089, 0.119)***	< 0.001	21.372
GGT	0 (reference)	0.145 (0.110, 0.180)**	0.311 (0.284, 0.339)***	< 0.001	30.626
AST/ALT	0 (reference)	− 0.114 (− 0.132, − 0.096)***	− 0.219 (− 0.234, − 0.204)***	< 0.001	66.897
Categorical outcome					
Abnormal ALT	1 (reference)	2.195 (1.735, 2.778)***	3.936 (3.079, 5.031)***	< 0.001	141.033
Abnormal AST	1 (reference)	1.178 (0.967, 1.434)	1.421 (1.158, 1.744)***	< 0.001	9.113
Abnormal ALP	1 (reference)	0.816 (0.543, 1.226)	1.421 (1.023, 1.974)*	0.008	6.896
Abnormal GGT	1 (reference)	1.503 (1.152, 1.962)**	2.336 (1.893, 2.883)***	< 0.001	33.852

Models were adjusted for age, gender, race, educational qualification, physical exercise, annual household income, smoking status, alcohol consumption, and batch (survey cycles). *P*_trend_ was tested by including non-abdominal obesity, pre-abdominal obesity, and abdominal obesity as 1, 2, and 3 (continuous variable) in models, respectively. *P < 0.05, **P < 0.01, ***P < 0.001.

Comparing with abdominal obesity, general obesity had a greater contribution to the effect of ALT (35.7% *vs* 30.3%), AST (2.4% *vs* 1.2%), GGT (38.0% *vs* 30.6%), and AST/ALT (73.9% *vs* 66.9%) levels, as well as the prevalence of abnormal ALT (147.3% *vs* 141.0%), abnormal AST (20.8% *vs* 9.1%), and abnormal GGT (44.1% *vs* 33.9%). However, it had a lower contribution to the level of ALP (16.8% *vs* 21.4%) and the prevalence of abnormal ALP (4.3% *vs* 6.9%).

### Combined associations of obesity and metabolically unhealthy status with liver injury

The combined effects of general obesity and metabolically unhealthy status on liver injury are presented in Table 4. With the subgroup characterized by non-general obesity and metabolically healthy status as a reference, the ALT levels of the other five subgroups were significantly increased (all *P* < 0.05), with the subgroup characterized by general obesity and metabolically unhealthiness having the greatest effect. Similar results were observed in the analyses of other liver enzyme levels and the prevalence of abnormal liver enzymes, whereas the AST/ALT ratio was the lowest in the subgroup characterized by general obesity and metabolically unhealthiness compared to the other subgroups. Additionally, whether in the metabolically healthy subgroup or the metabolically unhealthy subgroup, the levels of ALT, ALP, and GGT, as well as the prevalence of abnormal ALT and GGT were significantly increased from non-general obesity to overweight and finally to general obesity (all *P*_a_ < 0.05). On the other hand, regardless of being in the non-general obesity, the overweight, or the general obesity subgroup, the levels of ALT, ALP, and GGT, as well as the prevalence of abnormal ALT and GGT in the metabolically unhealthy subgroup were higher than that in metabolically healthy subgroup (all *P*_b_ < 0.05). The combined effect of abdominal obesity and being metabolically unhealthy on the liver injury was consistent with the above results (Table 5).

**Table 4:** Associations of combination of general obesity and metabolically unhealthy status with liver injury.

Indicators of liver injury	β/OR (95% *CI*)		*P*_b_
	Metabolically healthy status	Metabolically unhealthy status	
Continuous outcome			
ALT			
Non-general obesity	0 (reference)	0.065 (0.037, 0.092)***	< 0.001
Overweight	0.101 (0.071, 0.130)***	0.210 (0.184, 0.236)***	< 0.001
General obesity	0.198 (0.158, 0.238)***	0.315 (0.290, 0.341)***	< 0.001
*P*_a_	< 0.001	< 0.001	
AST			
Non-general obesity	0 (reference)	0.019 (0.001, 0.037)*	0.041
Overweight	0.012 (− 0.007, 0.031)	0.033 (0.013, 0.053)**	0.091
General obesity	0.004 (− 0.025, 0.033)	0.052 (0.034, 0.070)***	< 0.001
*P*_a_	0.726	< 0.001	
ALP			
Non-general obesity	0 (reference)	0.075 (0.052, 0.097)***	< 0.001
Overweight	0.059 (0.036, 0.083)***	0.103 (0.084, 0.123)***	< 0.001
General obesity	0.091 (0.065, 0.117)***	0.146 (0.126, 0.167)***	< 0.001
*P*_a_	< 0.001	< 0.001	
GGT			
Non-general obesity	0 (reference)	0.139 (0.104, 0.174)***	< 0.001
Overweight	0.108 (0.067, 0.150)***	0.283 (0.246, 0.319)***	< 0.001
General obesity	0.256 (0.207, 0.305)***	0.450 (0.418, 0.482)***	< 0.001
*P*_a_	< 0.001	< 0.001	
AST/ALT			
Non-general obesity	0 (reference)	− 0.046 (− 0.064, − 0.027)***	< 0.001
Overweight	− 0.089 (− 0.110, − 0.067)***	− 0.177 (− 0.194, − 0.160)***	< 0.001
General obesity	− 0.194 (− 0.223, − 0.164)***	− 0.263 (− 0.280, − 0.247)***	< 0.001
*P*_a_	< 0.001	< 0.001	
Categorical outcome			
Abnormal ALT			
Non-general obesity	1 (reference)	1.479 (1.066, 2.051)*	0.020
Overweight	1.765 (1.279, 2.437)***	3.082 (2.348, 4.045)***	0.002
General obesity	2.829 (1.941, 4.122)***	4.945 (3.872, 6.315)***	0.002
*P*_a_	< 0.001	< 0.001	
Abnormal AST			
Non-general obesity	1 (reference)	1.546 (1.163, 2.056)**	0.003
Overweight	1.270 (0.957, 1.686)	1.629 (1.231, 2.155)***	0.172
General obesity	1.240 (0.840, 1.830)	2.333 (1.836, 2.966)***	< 0.001
*P*_a_	0.383	< 0.001	
Abnormal ALP			
Non-general obesity	1 (reference)	2.121 (1.335, 3.369)**	0.002
Overweight	1.346 (0.715, 2.536)	1.725 (1.079, 2.758)*	0.401
General obesity	1.530 (0.787, 2.974)	2.435 (1.540, 3.852)***	0.180
*P*_a_	0.208	0.317	
Abnormal GGT			
Non-general obesity	1 (reference)	2.071 (1.439, 2.981)***	< 0.001
Overweight	1.439 (0.923, 2.243)	2.899 (2.077, 4.046)***	< 0.001
General obesity	2.248 (1.434, 3.524)***	4.138 (3.037, 5.639)***	0.003
*P*_a_	0.004	< 0.001	

Models were adjusted for age, gender, race, educational qualification, physical exercise, annual household income, smoking status, alcohol consumption, and batch (survey cycles). *P*_a_ was tested by including non-general obesity, overweight, and general obesity as 1, 2, and 3 (continuous variable) in metabolically healthy group and metabolically unhealthy group, respectively.* P*_b_ was tested by including metabolically healthy status and metabolically unhealthy status as 1 and 2 (continuous variable) in non-general obesity group, overweight group, and general obesity group, respectively. *P < 0.05, **P < 0.01, ***P < 0.001.

**Table 5:** Associations of combination of abdominal obesity and metabolically unhealthy status with liver injury.

Indicators of liver injury	β/OR (95% *CI*)		*P*_b_
	Metabolically healthy status	Metabolically unhealthy status	
Continuous outcome			
ALT			
Non-abdominal obesity	0 (reference)	0.075 (0.045, 0.105)***	< 0.001
Pre-abdominal obesity	0.097 (0.066, 0.128)***	0.200 (0.168, 0.232)***	< 0.001
Abdominal obesity	0.183 (0.149, 0.216)***	0.306 (0.279, 0.334)***	< 0.001
*P*_a_	< 0.001	< 0.001	
AST			
Non-abdominal obesity	0 (reference)	0.023 (0.002, 0.044)*	0.034
Pre-abdominal obesity	0.008 (− 0.015, 0.031)	0.030 (0.008, 0.053)**	0.171
Abdominal obesity	0.005 (− 0.019, 0.029)	0.044 (0.023, 0.065)***	< 0.001
*P*_a_	0.963	0.038	
ALP			
Non-abdominal obesity	0 (reference)	0.070 (0.046, 0.095)***	< 0.001
Pre-abdominal obesity	0.015 (− 0.008, 0.038)	0.075 (0.051, 0.099)***	< 0.001
Abdominal obesity	0.100 (0.074, 0.125)***	0.147 (0.125, 0.168)***	< 0.001
*P*_a_	< 0.001	< 0.001	
GGT			
Non-abdominal obesity	0 (reference)	0.158 (0.119, 0.197)***	< 0.001
Pre-abdominal obesity	0.130 (0.085, 0.176)***	0.274 (0.231, 0.317)***	< 0.001
Abdominal obesity	0.221 (0.184, 0.257)***	0.433 (0.401, 0.465)***	< 0.001
*P*_a_	< 0.001	< 0.001	
AST/ALT			
Non-abdominal obesity	0 (reference)	− 0.052 (− 0.071, − 0.033)***	< 0.001
Pre-abdominal obesity	− 0.089 (− 0.112, − 0.066)***	− 0.170 (− 0.192, − 0.148)***	< 0.001
Abdominal obesity	− 0.178 (− 0.201, − 0.154)***	− 0.262 (− 0.280, − 0.245)***	< 0.001
*P*_a_	< 0.001	< 0.001	
Categorical outcome			
Abnormal ALT			
Non-abdominal obesity	1 (reference)	1.567 (1.095, 2.243)*	0.015
Pre-abdominal obesity	2.129 (1.510, 3.003)***	3.139 (2.255, 4.370)***	0.075
Abdominal obesity	2.926 (2.002, 4.276)***	5.575 (4.044, 7.685)***	< 0.001
*P*_a_	< 0.001	< 0.001	
Abnormal AST			
Non-abdominal obesity	1 (reference)	1.497 (1.141, 1.965)**	0.004
Pre-abdominal obesity	1.269 (0.925, 1.741)	1.556 (1.180, 2.053)**	0.382
Abdominal obesity	0.998 (0.695, 1.435)	1.978 (1.528, 2.562)***	< 0.001
*P*_a_	0.937	0.014	
Abnormal ALP			
Non-abdominal obesity	1 (reference)	2.949 (1.742, 4.992)***	< 0.001
Pre-abdominal obesity	1.058 (0.455, 2.460)	1.902 (1.158, 3.125)*	0.137
Abdominal obesity	2.570 (1.524, 4.333)***	2.928 (1.819, 4.713)***	0.651
* P*_a_	< 0.001	0.692	
Abnormal GGT			
Non-abdominal obesity	1 (reference)	2.334 (1.553, 3.509)***	< 0.001
Pre-abdominal obesity	1.549 (0.936, 2.564)	2.973 (2.043, 4.327)***	0.004
Abdominal obesity	2.135 (1.385, 3.292)***	4.261 (2.964, 6.127)***	< 0.001
*P*_a_	0.012	< 0.001	

Models were adjusted for age, gender, race, educational qualification, physical exercise, annual household income, smoking status, alcohol consumption, and batch (survey cycles). *P*_a_ was tested by including non-abdominal obesity, pre-abdominal obesity, and abdominal obesity as 1, 2, and 3 (continuous variable) in metabolically healthy group and metabolically nhealthy group, respectively. Pb was tested by including metabolically healthy status and metabolically unhealthy status as 1 and 2 (continuous variable) in non-general obesity group, overweight group, and general obesity group, respectively. *P <0.05, **P <0.01, ***P <0.001.

### The effect of weight change on liver injury in retrospective analysis

Table 6 reveals that 1-year and 10-year weight changes were significantly associated with liver enzyme levels as well as the prevalence of abnormal liver enzymes. Each 1-kg increase in 1-year weight change was associated with increased levels of ALT, AST, ALP, and GGT by 0.53% (0.42%, 0.65%), 0.18% (0.09%, 0.27%), 0.14% (0.06%, 0.23%), and 0.39% (0.24%, 0.54%), respectively. Moreover, it was associated with a heightened risk of abnormal ALT and AST by 3.2% (2.2%, 4.2%) and 1.5% (0.5%, 2.5%), respectively. Meanwhile, the estimated changes of ALT, AST, ALP, and GGT levels, along with the risk of abnormal ALT and AST significantly increased with the elevated quartiles of 1-year weight change (*P*_trend_ < 0.05). In contrast, 1-year weight change was negatively associated with the AST/ALT ratio (*P* and *P*_trend_ < 0.05). Similarly, each 1-kg increase in 10-year weight change was associated with increased levels of ALT, AST, ALP, and GGT by 0.54% (0.46%, 0.62%), 0.11% (0.05%, 0.17%), 0.18% (0.12%, 0.25%), and 0.71% (0.59%, 0.82%), respectively, as well as increased risk of abnormal ALT, AST, and GGT by 3.2% (2.5%, 3.9%), 1.5% (0.7%, 2.3%), and 1.9% (1.3%, 2.6%), respectively. With the increasing quartiles of 10-year weight change, the levels of ALT, AST, ALP, and GGT, as well as the prevalence of abnormal ALT, AST, and GGT gradually increased (*P*_trend_ < 0.05).

**Table 6:** Associations of weight change with liver injury.

Weight change/liver injury	β/OR (95% *CI*) by continuous weight change	β/OR (95% *CI*) by quartiles of weight change				*P*_trend_
		Q1	Q2	Q3	Q4	
1 year Continuous outcome						
ALT	0.0053 (0.0042, 0.0065)***	0 (reference)	− 0.021 (− 0.043, 0.002)	0.038 (0.012, 0.063)**	0.113 (0.089, 0.137)***	< 0.001
AST	0.0018 (0.0009, 0.0027)***	0 (reference)	− 0.009 (− 0.025, 0.007)	0.026 (0.006, 0.045)*	0.035 (0.018, 0.053)***	< 0.001
ALP	0.0014 (0.0006, 0.0023)**	0 (reference)	− 0.029 (− 0.046, − 0.011)**	− 0.015 (− 0.034, 0.004)	0.026 (0.008, 0.044)**	0.002
GGT	0.0039 (0.0024, 0.0054)***	0 (reference)	− 0.069 (− 0.101, − 0.036)***	− 0.004 (− 0.038, 0.029)	0.064 (0.030, 0.097)***	< 0.001
AST/ALT	− 0.0035 (− 0.0042, − 0.0028)***	0 (reference)	0.012 (− 0.004, 0.027)	− 0.012 (− 0.028, 0.004)	− 0.078 (− 0.093, − 0.062)***	< 0.001
Categorical outcome						
Abnormal ALT	1.032 (1.022, 1.042)***	1 (reference)	0.823 (0.653, 1.037)	1.273 (0.989, 1.639)	1.812 (1.470, 2.234)***	< 0.001
Abnormal AST	1.015 (1.005, 1.025)**	1 (reference)	0.723 (0.596, 0.877)**	0.999 (0.793, 1.259)	1.281 (1.046, 1.570)*	0.003
Abnormal ALP	1.005 (0.984, 1.026)	1 (reference)	0.843 (0.605, 1.175)	0.812 (0.571, 1.156)	1.089 (0.793, 1.496)	0.699
Abnormal GGT	1.007 (0.998, 1.016)	1 (reference)	0.758 (0.609, 0.943)*	0.958 (0.766, 1.197)	1.088 (0.891, 1.329)	0.158
10 years						
Continuous outcome						
ALT	0.0054 (0.0046, 0.0062)***	0 (reference)	0.025 (0.004, 0.047)*	0.095 (0.073, 0.118)***	0.201 (0.173, 0.229)***	< 0.001
AST	0.0011 (0.0005, 0.0017)***	0 (reference)	0.017 (0.001, 0.033)*	0.020 (0.001, 0.039)*	0.045 (0.024, 0.065)***	< 0.001
ALP	0.0018 (0.0012, 0.0025)***	0 (reference)	− 0.034 (− 0.054, − 0.014)*	0.002 (− 0.019, 0.022)	0.064 (0.044, 0.083)***	< 0.001
GGT	0.0071 (0.0059, 0.0082)***	0 (reference)	0.002 (− 0.031, 0.036)	0.079 (0.038, 0.120)***	0.247 (0.207, 0.287)***	< 0.001
AST/ALT	− 0.0043 (− 0.0049, − 0.0038)***	0 (reference)	− 0.008 (− 0.026, 0.010)	− 0.075 (− 0.092, − 0.058)***	− 0.156 (− 0.174, − 0.139)***	< 0.001
Categorical outcome						
Abnormal ALT	1.032 (1.025, 1.039)***	1 (reference)	0.942 (0.687, 1.292)	1.584 (1.239, 2.025)***	2.876 (2.209, 3.744)***	< 0.001
Abnormal AST	1.015 (1.007, 1.023)***	1 (reference)	1.057 (0.833, 1.340)	1.206 (0.946, 1.536)	1.574 (1.213, 2.042)***	< 0.001
Abnormal ALP	1.008 (0.992, 1.024)	1 (reference)	0.681 (0.499, 0.929)*	0.596 (0.421, 0.845)**	1.265 (0.913, 1.752)	0.237
Abnormal GGT	1.019 (1.013, 1.026)***	1 (reference)	0.881 (0.681, 1.140)	1.006 (0.786, 1.288)	1.732 (1.393, 2.153)***	< 0.001

Models were adjusted for age, gender, race, educational qualification, physical exercise, annual household income, smoking status, alcohol consumption, and batch (survey cycles). Ptrend was tested by including quartiles of weight change as 1, 2, 3, and 4 (continuous variable) in models, respectively. *P <0.05, **P <0.01, ***P <0.001.

## Discussion

In the present study, we found that both general obesity and abdominal obesity were positively associated with the indicators of liver injury, with general obesity having a higher contribution. Metabolic abnormalities and obesity had a combined effect on liver injury. Both short-term and long-term weight gain/loss were associated with increased/decreased risk of liver injury.

We used liver enzyme levels as indicators of liver injury. As obtaining liver biopsy is nearly impossible in large-scale general population-based epidemiological studies, blood biomarkers are commonly used. It is noteworthy that liver enzymes, even when released by a small proportion of damaged hepatocytes, can lead to a substantial elevation in serum liver enzyme levels. Consequently, according to the clinical guidelines established by the American College of Gastroenterology (ACG), these enzymes should be considered as indicators of liver injury^28,29^. Previous studies have commonly employed ALT, AST, ALP, and GGT as markers to evaluate liver injury^30,31^.

Obesity is a recognized risk factor for liver injury. Liver injuries, including NAFLD, can be caused by excessive fat accumulation, which induces an oversupply of fatty acids in the liver, hepatocyte injury, and chronic low-grade inflammation^32,33^. BMI is the most widely used crude measure of general obesity. Compared with BMI, waist circumference is strongly associated with abdominal fat distribution and is a better index for abdominal obesity. Dose–response analysis suggested that higher BMI is an independent and dose-dependent risk factor for fatty liver^34^. Population-based study found that WC to be independently associated with liver disease^35^. These studies suggest that both general obesity and abdominal obesity pose a risk for liver injury. Although previous studies have suggested that WC may be more strongly associated with an elevated risk of liver cancer compared to BMI^11^, the contribution of the two obesity types to liver injury has rarely been reported. Our study, on the other hand, found that general obesity had a larger impact on the association with liver enzyme levels and the prevalence of abnormal liver enzymes compared to abdominal obesity. Nonetheless, it is important to note that further validation of these findings is warranted through larger prospective studies.

Metabolic disorders, including insulin resistance, dyslipidemia, and hypertension, are important driving factors on liver injury. Insulin resistance is often associated with chronic low-grade inflammation and abnormal fat metabolism, which leads to the release of numerous molecular mediators from immune cells and adipocytes, and ultimately contributes to liver injury, liver disease progression, and liver repair disorders^36,37^. Hypertension may induce liver injury and hepatic fibrosis through decreased interleukin-10-mediated or heme oxygenase-1-induced anti-inflammatory mechanisms^38^. Although obesity is usually associated with metabolic disorders, it is possible for obese individuals to be metabolically healthy. A large cohort study of metabolically healthy population suggested that obesity was strongly and progressively associated with an increased incidence of NAFLD^39^, indicating that obesity is an independent risk factor for liver injury. In our study, we found that obesity and metabolic health status are independent of each other in the associations with liver enzyme levels and abnormal liver enzyme, furthermore, they had combined effects on liver injury.

Existing evidence indicates that weight gain is an important risk factor for liver injury in general population, and weight loss has shown potential to ameliorate liver injury. A randomized controlled trial showed that weight loss through diet significantly reduced the levels of liver enzymes, including ALT and GGT^40^. In our study, we investigated the effect of weight change on liver injury parameters and found that both short- and long-term weight change were associated altered risk for liver injury, with weight gain associated with increased risk and weight loss associated with lower risk. Our findings are consistent with previous studies.

The present study utilized data from a large population and analyzed association of different obesity phenotypes as well as and metabolic health status with indicators of liver injury. Our findings provide new insights into the complicated interactions between obesity, metabolism and the risk of liver injury. Nevertheless, there are some limitations in our study. Firstly, it is a cross-sectional association study, and a causal effect of these risk factors still needs to be confirmed in prospective cohort studies. Secondly, even if some covariates were adjusted in our statistical analyses, other variables such as genes that are associated with outcome variables were not available in NHANES datasets. Finally, it should be noted that liver enzymes have a relatively short half-life in the systemic circulation, typically spanning only a few days. Therefore, in order to obtain a more accurate representation of liver injury, it is recommended to perform liver chemical examinations on at least two occasions, with a minimum interval of six months between each assessment^41,42^.

## Conclusion

In the present study, we analyzed a large population dataset and established that both obesity and metabolic health status act as independent risk factors for liver injury. Moreover, these factors exhibit combined effects, further exacerbating the risk. Additionally, our findings revealed a positive association between weight fluctuations and the likelihood of developing liver injury. These results underscore the urgent necessity for increased focus on liver health among adults with metabolically unhealthy obesity and emphasize the significance of weight loss interventions in improving liver injury outcomes.

## Data Availability

The datasets analysed during the current study are available at NHANES website https://www.cdc.gov/nchs/nhanes/.
